# Intelligence

**Published:** 1808-03

**Authors:** 


### INTELLIGENCE.

The experiments of Mr. Davy on the Alkalies have been repeated before the Askesian and Mineralogical Societies, by Mr. Pepys, with a very large galvanic apparatus, consisting of one hundred and twenty pair of plates of 36 inches surface each, containing nearly seven hundred weight of copper and zinc. The solid caustic pot-ash was used, slightly moistened by the breath. The metalloid metalloid obtained was highly inflammable, swam in rectified naptha, but was with difficulty separated from the pot-ash, in which it is plentifully imbedded, after being exposed to the galvanic action. Water being dropped upon it, the particles explode similarly to grains of gunpowder thrown into the fire. The metalloid obtained from soda is not so highly inflammable, and can therefore be collected more easily. A globule, about the size of a small tare, being thrown on paper moistened, instantly became apparently red hot, and running off the surface of the water, fell luminously through the air. Mr. Allen has also repeated the experiments of Mr. Davy, and obtained both the metalloids by four troughs of fifty pairs, each of 16 inches surface.

---

A Paper on Oxalic Acid, by Dr. Thornton, of Edinburgh, was lately read before the Royal Society; containing the results of a great variety of experiments made to ascertain the relative qualities of the constituent parts of this acid, on a principle of calculation laid down by this able chemist in the last edition of his excellent System of Chemistry.

---

A complete Mammoth has lately been found (though not alive) in a state of perfect preservation, on the borders of the Frozen Ocean. It was discovered by Schoumachoff, a Tungoose Chief, in the autumn of 1799, in the midst of a rock of ice; but it was not till the fifth year after finding it, that the ice had melted sufficiently to disengage the Mammoth, when it fell over on its side on a bank of sand. Schoumachoff then cut off the tusks, which he bartered for goods with a Russian merchant to the value of fifty rubles (eleven pounds five shillings). He then left the carcase to be devoured by bears and wolves; but, previously to which, he had a rude drawing made of it, which represents it with pointed ears, very small eyes, horses hoofs, and a bristly mane, extending along the whole of its back. — In 1806, Mr. Michael Adams, of Petersburgh, hearing of the circumstance, repaired to the spot, where he found the skeleton entire, one of the fore feet excepted, though nearly stripped of its flesh. The vertebræ, from the head to the os coccygis, one of the shoulder blades, the pelvis, and the remaining three extremities, were still held firmly together by the ligatures of the joints, and by strips of skin and flesh. The head was covered with a dry skin. One of the ears, well preserved, was furnished with a tuft of bristles. These parts could not avoid receiving some injury, during their removal to Petersburgh, a distance of 6875 miles; the eyes, however, are preserved, and the pupil of the left eye is still distinguishable. The tip of the under lip was eaten away, and the upper being destroyed, the teeth were exposed. The brain, still within the cranium, appeared quite dry. The parts least damaged, were one of the fore feet and one of the hind; these were still covered with skin, and ha[illegible] the sole attached to them. According to the Tungoose chief, the animal was so corpulent and well fed, that its body hung down below the knee joints. It

It was a male, but had neither tail nor trunk. From the structure of the os coccygis, however, Mr. Adams is persuaded that it had a thick short tail. Schoumachoff always persisted in asserting, that he never saw any appearance of a proboscis; and it does not appear probable, that his rude draughtsman would have omitted such a striking feature if there had been one. The skin, (three fourths of which is in the possession of Mr. Adams,) was of a deep grey colour, and covered with reddish hair and black bristles. More than 40lbs. weight of them, that had been trodden into the ground by the bears, were collected, and many of them were 2 feet 4 inches long. The head weighs 460lbs.; the two horns, each of which is $9\frac{1}{2}$ feet long, weigh 400lbs.; and the entire animal measured $10\frac{1}{2}$ feet high by $16\frac{1}{2}$ feet long. The tusks are curved in the direction opposite to those of the elephant, bending toward the body of the animal. Mr. Adams adds that he found a great quantity of amber on the shores.

---

Mr. Hill, of Hinckley, is preparing a work on those Diseases of the Bones which produce Distortions of the Spine and Limbs; in which, the medical, surgical, and mechanical modes of treatment will be considered, and the latter mode illustrated by plates.

---

Dr. Clarke and Mr. Clarke will begin a Course of their Lectures on Midwifery, and the Disorders of Women and Children, on Tuesday, March the 22d. The Lectures are read every day at the house of Mr. Clarke, No. 10, Upper John Street, Golden Square, from a quarter past ten o'clock in the morning till a quarter past eleven, for the convenience of Students attending the Hospitals.

Dr. Reid will commence his next Course of Lectures on the Theory and Practice of Medicine, on Monday the 7th of March, at ten o'clock in the morning; at which hour the Course will be continued, on Mondays, Wednesdays, and Fridays, during three months, at his house, Grenville-street, Brunswick-square.

